# Development and Validation of the Motivations for Selection of Medical Study (MSMS) Questionnaire in India

**DOI:** 10.1371/journal.pone.0164581

**Published:** 2016-12-20

**Authors:** Sonu Goel, Federica Angeli, Neetu Singla, Dirk Ruwaard

**Affiliations:** 1 School of Public Health, Post Graduate Institute of Medical Education and Research, Chandigarh, India; 2 Department of Health Services Research, Faculty of Health, Medicine and Life Sciences, Maastricht University, Maastricht, The Netherlands; Iran University of Medical Sciences, ISLAMIC REPUBLIC OF IRAN

## Abstract

**Background and Objective:**

Understanding medical students’ motivation to select medical studies is particularly salient to inform practice and policymaking in countries—such as India—where shortage of medical personnel poses crucial and chronical challenges to healthcare systems. This study aims to develop and validate a questionnaire to assess the motivation of medical students to select medical studies.

**Methods:**

A Motivation for Selection of Medical Study (MSMS) questionnaire was developed using extensive literature review followed by Delphi technique. The scale consisted of 12 items, 5 measuring intrinsic dimensions of motivations and 7 measuring extrinsic dimensions. Exploratory factor analysis (EFA), confirmatory factor analysis (CFA), validity, reliability and data quality checks were conducted on a sample of 636 medical students from six medical colleges of three North Indian states.

**Results:**

The MSMS questionnaire consisted of 3 factors (subscales) and 8 items. The three principal factors that emerged after EFA were the scientific factor (e.g. research opportunities and the ability to use new cutting edge technologies), the societal factor (e.g. job security) and the humanitarian factor (e.g. desire to help others). The CFA conducted showed goodness-of-fit indices supporting the 3-factor model.

**Conclusion:**

The three extracted factors cut across the traditional dichotomy between intrinsic and extrinsic motivation and uncover a novel three-faceted motivation construct based on scientific factors, societal expectations and humanitarian needs. This validated instrument can be used to evaluate the motivational factors of medical students to choose medical study in India and similar settings and constitutes a powerful tool for policymakers to design measures able to increase selection of medical curricula.

## Introduction

India is facing an acute shortage of medical practitioners in rural and regional areas, especially in North India [1, 2]. The shortage has become more critical over the last two decades. The migration of physicians from developing to developed countries and intra-country disparities between urban and rural regions are argued as the main reasons [3–7].There have been considerable efforts from the Government of India to address these shortages through a wide range of programs and policies targeting medical students [8]. The ‘High Level Expert Group for Universal Health Coverage’ constituted by the Planning Commission has set the minimum doctor-to-population ratio to 1:1,000 [9]. Currently, India counts only 57 physicians for 100,000 people or 1 physician for over 1,700 people, indicating that more qualified medical professionals are needed [10]. For 70% of the Indian rural population, the patient-physician ratio is extremely low and amounts to a mere 1 physician for over 2,564 inhabitations [2]. The proposed ratio of 1:1,000 can be achieved only if more students will opt for medical study and remain in India to practice the profession. Although the Medical Council of India (MCI) reports educating 52,105 doctors each year [11], almost one third of them leave India for residency training and/or practice abroad [12].

Globally, the medical profession is one of the most reputed and highly paid professions. In the Indian context, medical professionals are highly respected and are associated with high social status. This cultural trait affects the students in the selection of the medical profession after post matriculation examination. Each individual has a different reason for choosing the medical field, although factors such as interest in the medical field, good job opportunities, desire to serve others, medical background of the parents and many more are commonly reported motivators [13]. In India the reasons for selecting the medical profession vary across people but also across geographic locations. Understanding the main motivational items that influence students to opt for medical studies is therefore complex. Motivation of students to enter medical study is an important issue because it affects the number of physicians graduating each year from medical schools and therefore the services they can ultimately provide within the national healthcare system.

Several studies have been conducted worldwide to determine the factors underpinning students’ motivation to opt for medical study [14–32]. However, only very few of them have been performed in India [10, 33, 34, 35]. These studies have focused on very limited items of motivation and in different Indian states. Understanding the motivating factors underpinning the choice for medical study in the Indian context is crucial to frame appropriate policies and organizational strategies to counteract the acute shortage of medical personnel and its sharply uneven distribution across urban and rural areas.

An array of questionnaires has been developed to identify the main reasons why medical students selected a medical study [36–41]. However, these tools have been constructed and validated in other countries, such as strength of motivation for medical school (SMMS) questionnaire by Kusurkar R. et al. [41] in the Netherlands; a scale to measure intrinsic and extrinsic motivators to study medicine by Agyei-Baffour et al. [13] in Ghana, Academic Motivation Scale (AMS) by Vallerand et al. [36, 37, 38] in Canada, and Maslach Burnout Inventory-Student Survey (MBI-SS) [39]. Applying these instruments in culturally different contexts, such as India, could lead to bias and unreliable findings [13, 36–41].

To the best of our knowledge, studies in India have either used scales validated in western settings or used non-validated instruments to measure medical student’s motivation to select medical studies [13, 36–41]. None of the studies has comprehensively studied the factors in Indian settings. This hinders the valid and robust measurement of students’ motivations to select medical studies in India. There is hence a need for a valid and reliable tool to measure reasons that motivated medical students to choose medical study suited for use in India and perhaps other developing countries. To tackle this important gap, we decided to develop a valid and reliable instrument for measuring the choice of medical students to study Bachelor of Medicine, Bachelor of Surgery (MBBS) in Indian settings.

## Methods

The study was ethically approved from the Institute’s Ethical Committee, Post Graduate Institute of Medical Education and Research (PGIMER), Chandigarh (PGI/IEC/2012/810-1 P-154). The permission from the Principals of all medical colleges was obtained prior to the study. The students provided written informed consent to participate in the study. All the students voluntarily participated in the study after being informed about the objectives of the study and assured about anonymity of the responses.

### Item generation

#### Literature review

Because the socio-cultural peculiarities of the Indian context and the socio-cultural heterogeneity between Indian states could undermine the validity of pre-validated measurement tools, a structured questionnaire was developed for the purpose of this study (S1 File). The questionnaire was based on extensive literature search that was carried out with the purpose to identify the perceptions of medical students to enter in medical study. The search was carried out by two researchers independently in PubMed, IndMED, Directory of Open Access journals and Google scholar. Medical Subject Headings (MeSH) and free-text terms “motivation, motivator, motivate” AND “selection, choose” AND “medical students, interns, medical school, medical study” have been used. Search terms and keywords were altered as per specification of individual databases. In addition, a manual search of articles in journals held in the library of the PGIMER was done. Accordingly, thousands of articles were identified from various search engines. After removing duplication and screening of titles, abstracts and full text, 43 relevant articles on the subject were selected.

#### Questionnaire development

Delphi technique was used in the process. A Delphi technique is a practical approach to achieve a consensus among a group of experts. It is based on the assumption that group opinion has a greater validity than an individual opinion [42].

Two rounds of Delphi were conducted. In the first round, the initial versions of the questionnaire was presented to technical experts (n = 7), teachers of medical colleges (n = 10) and doctors (n = 5) of clinical departments in health care facilities of Chandigarh city. Discussions were held on various dimensions of motivations to select a medical study. The first author then built consensus on the items to be included in motivations for selection of the medical study by students. The second round was held two weeks later. The first author shared the responses of various experts among themselves with the purpose of clarity of responses and for the finalization of questionnaire.

#### Pilot testing of questionnaire

The questionnaire was pretested on 20 students of a government medical college (not participating in the study). To examine whether field investigators correctly administered the questionnaire, five interviews were audio recorded. The principal investigator found no problem in administration of the instrument and found that questions were correctly interpreted by students. No item was deleted or modified. The structure of the questionnaire remained the same after this stage (i.e.12 items).

### Item reduction

#### Data collection

***Study site*:** Three North Indian states, namely Himachal Pradesh, Punjab and Haryana, were selected as study site. Two government medical colleges from each state were considered for data collection. The students in medical colleges were selected for admission by a common state medical entrance test. In India, medical education consists of 5 years of medical study followed by one year of clinical training (known as ‘internship’) in a rural or urban hospital attached to medical college. The Medical Council of India (MCI) is the statutory regulatory and registration authority for medical education and practitioners in India. The MCI has the authority to cancel the registration and recognition of any medical college in the country if the college does not comply with its guidelines.

***Study population*:** The data collection was conducted during the period from November 2014 to January 2016 in six Government medical colleges (two from each state). The data was collected by two researchers who were trained in administering the questionnaire by the first author, which helped to standardize the administration of the instrument. All final year medical (MBBS) students of the college were invited to participate in the study, resulting in 636 participants. The sample size is appropriate for the study as most studies of questionnaire’s validation in social sciences use 5 to 10 respondents per questionnaire item for factor analysis [43,44]. Students were asked to rate the items on a 5-point Likert scale where 1 represents “very unlikely to select”, 2 represents “unlikely to select”, 3 represents “not Sure”, 4 represents “likely to select” and 5 represents “very likely to select”. The questionnaire was handed out and collected after completion. To preserve the anonymity and confidentiality of participants, the participants were asked to place the filled questionnaire in a sealed box.

#### Content validity

A content validity index, as proposed by Lynn [45], was defined by an independent group of experts (different from the original panel included in the Delphi study). The expert group was asked to assess the content of each item generated on a five-point Likert scale. The assessment was based on the appropriateness, comprehensibility and clarity of phrasing of each item.

#### Data quality

Data quality was assessed by checking the percentage of missing data, extent of ceiling and floor effects and corrected item-to-total correlation. The ceiling and floor effects usually happen when the score for an item in the scale are rated very high and low by respondents respectively. Corrected item-to- is the correlation between each item and the total score from the questionnaire and all the items should correlate with the total for a reliable scale. Items were eliminated if: the missing response rate of an item was more than 10%; the floor and ceiling effect of an item was between 1% and15%; and items had a correlation of less than 0.30 with the total scale score (corrected item-to-total correlation) [46].

#### Validation and reliability

The main outcome of interest was the validation of MSMS scale and extraction of relevant motivation factors for MBBS students to select medical study. Students were identified as having strong intrinsic motivation if two or more of their motivational items (out of 5) were strongly intrinsic (i.e. they responded as 4 or 5 on 5 point Likert scale) and having strong extrinsic motivation if two or more of their motivational items (out of 7) were strongly extrinsic [13]. Summary statistics for socio-demographic variables as well as for the list of twelve items were calculated.

In the context of construct validity, exploratory factor analysis (EFA) with varimax rotation was applied on the MSMS list of items to group items with similar characteristics together (extraction of factor structure), which further gives a small list of factors/ subscales capable of explaining most of the variance. Kaiser-Meyer-Olkin (KMO) test was used to check sampling adequacy which should be greater than 0.5 for a satisfactory factor analysis to proceed [47]. Bartlett's test was applied to check the strength of the relationship among items. The criterion of eigenvalue or characteristic root (Eigenvalue) ≥1 was used for defining the number of the factors that were kept [48–50]. Scree plot, a graphic representation of eigenvalues, suggests the number of the essential factors to be retained. After the rotation each item was loaded in one or another factor. Items with factor loading greater than 0.4 were retained [50]. Cronbach’s alpha for internal consistency was determined for establishing the reliability of the subscales. Convergent and discriminant validity was checked using Spearman correlation. The value of a correlation coefficient of greater than 0.40 between an item and its own scale is regarded as an adequate evidence of convergent validity. Discriminant validity is supported whenever a correlation between an item and its hypothesised scale is higher than its correlation with the other scales. A scaling success is counted if the item to own-scale correlation is significantly higher than the correlations of the item to other scale [51].

EFA was followed by Confirmatory factor analysis (CFA) for validating the underlying structure of MSMS scale on prior empirical and theoretical grounds. CFA is a particular case of structural equation modeling (SEM) which consists of collecting data in order to confirm that a factor is defined according to the theoretical approach the researcher uses as a starting point. Then, the model will serve to represent, in a reasonably good way, how the observed variables are interconnected [52]. We calculated goodness of fit of CFA model using widely accepted indices: root mean square error of approximation (RMSEA), comparative fit index (CFI) and GFI (Goodness-of-fit index). The cut off values of acceptable model fit cited by Leach et al. [53] are CFI and GFI values greater than .93 and RMSEA below .08. Data was entered and analyzed using Statistical Package for Social Sciences version- 16 (SPSS IBM, New York, NY, USA). To ensure accuracy of data, double data entry was done. SPSS/Amos 22 software was used for CFA.

**Fig 1:**
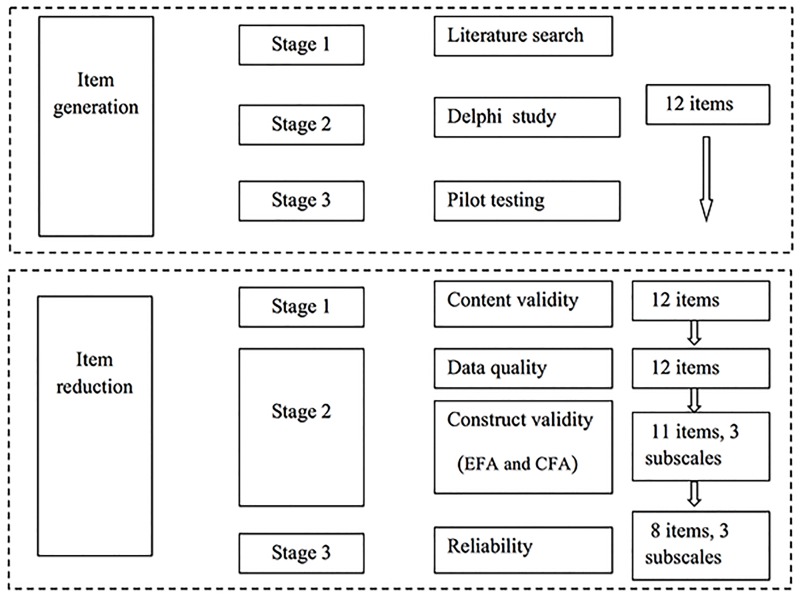
Steps for development and validation of MSMS.

The steps for the development and validation of the questionnaire are shown in Fig 1.

## Results

### Item generation

Based on the literature review, a framework on various dimensions of motivation to select medical study by medical students was developed, resulting in a 12-item scale named the Motivations for Selection of Medical Study (MSMS) scale. During the two rounds of Delphi, the experts revisited their responses along with responses of other experts involved in the process, refined wording and content of questions to reach a consensus.

In pilot testing, no potential problems were found in the administration of the instrument by the field investigators. The structure of the questionnaire remained the same after this stage (i.e. twelve items).The questionnaire was divided into two parts: Part 1 was meant to assess the socio-demographic profile of the medical students while Part 2 investigated the items/reasons why they choose a medical study. Part 2 included a total of twelve items: five intrinsic items (desire to help others, desire to give back to your community or country, interest in medicine as a subject matter, inspiration by a role model and loss of loved ones) and seven extrinsic items (income of physician, job security and lifestyle, social status/prestige, proposed by parents, opportunities to travel and work internationally, ability to use new cutting edge technologies and research opportunities) for selection of a medical study.

### Item reduction

#### Results on study settings

***Demographics*:** The total sample consisted of 636 MBBS final year students (response rate = 100%) of both genders. Out of 636, a total of 297 (44.7%) were male with mean age 22.4 years (SD 2.03) and 339 (53.3%) were females with mean age 22.1 years (SD 1.53). A total of 405 (63.7%) students were born in rural areas and the majority of the students (80.8%) studied in urban areas before MBBS. The socio-demographic characteristics are presented in Table 1.

**Table 1:** Demographic characteristics of medical students.

Characteristics		Frequency (%)
**Age-group**	19–24	521 (81.9)
	24–29	111 (17.5)
	> 29	4 (0.6)
**Sex**	Male	297 (46.7)
	Female	339 (53.3)
**Caste**	General	467 (73.4)
	Others (SC/ST/OBC/ others)	169 (26.6)
**Religion**	Hindu	518 (81.4)
	Others(Christian, Sikh, Muslim, Jain and Buddhism)	118 (18.6)
**Marital status**	Married	12 (1.9)
	Unmarried	622 (97.8)
	Others	2 (0.3)
**Residential status**	Living alone	389 (61.2)
	Living with family	247 (38.8)
**Birth location**	Urban	405 (63.7)
	Rural	231 (36.3)
**Family background**	Urban	377 (59.3)
	Rural	259 (40.7)
**Location of premedical studies**	Urban	515 (81.0)
	Rural	121 (19.0)

#### Content validity and data quality

No item was deleted after the expert group review (Delphi technique). The content validity index of the item scale (12 items and 3 factors) was 0.75 which was acceptable according to conventional criteria [46]. As the researchers were available during the process of filling out the questionnaire missing data was very negligible. No floor or ceiling effects were observed.

#### Construct validity and reliability

Exploratory factor analysis (EFA) was carried out with varimax rotation. KMO measure scores 0.690, which indicates that the sample is adequate for factor analysis [54]. Bartlett's test of sphericity rejected null hypothesis at 0.05 level of significance (Bartlett's test significance<0.05) and ensures the relevance of factor analysis. Three factors (subscales) reporting eigenvalue ≥1 emerged, which together explained 51.53% of the total variance in the MSMS scale. Table 2 depicts the three factors with corresponding loadings of items.

**Table 2:** Loadings of items from the MSMS questionnaire on the three main factors.

	Factor 1	Factor 2	Factor 3
Ability to use new cutting edge technologies	**.845**	.112	.147
Opportunities to travel and work internationally	**.765**	.237	.033
Research opportunities	**.764**	-.073	.211
Loss of loved one	**.430**	.050	-.168
Inspiration by a role model	.311	.164	.224
Job security	-.015	**.817**	.010
Social status	-.050	**.757**	.202
High income	.298	**.666**	-.201
Proposed by parents	.205	**.452**	.039
Desire to help others	-.062	.025	**.829**
Desire to give back to community	.066	.056	**.759**
Interest in medicine as a subject	.144	-.007	**.608**

The three factors emerged out in our study were labeled as:

1. Scientific factor of motivation (Factor 1)
2. Societal factor of motivation (Factor 2)
3. Humanitarian factor of motivation (Factor 3)

***Factors explanation*:** Factor 1: Scientific factor of motivation. This factor measures the scientific reasons that motivate medical students to select medical study. It explains 23.73% of the total variance in the MSMS scale. *Ability to use new cutting edge technologies*, *opportunities to travel and work internationally*, *research opportunities* and *loss of a loved one* are four items substantially loading on this factor. Internal consistency of this subscale was checked by Cronbach’s alpha (Table 3) which was 0.699, but increases to .783 by dropping item *loss of a loved one*. So this item was eliminated and the final sub-scale is formed by three items.

**Table 3:** Cronbach’s alpha coefficient, convergent and discriminant validity for three subscales.

Factor	No of Items	Mean ± SD	Alpha	Convergent validity		Discriminant validity	
				Range of correlation	Scaling success	Range of correlation	Scaling success
**Scientific**	3	3.223 ± 1.366	0.783	0.563–0.735	3/3	0.072–0.280	6 /6
**Societal**	3	3.653 ± 1.232	0.660	0.436–0.555	3/3	0.054–0.294	6/6
**Humanitarian**	2	4.062 ± 0.859	0.699	0.542	2/2	0.054–0.294	4/4

Factor 2: Societal factor of motivation. This factor explains the social variables that motivate medical students to select medical study. It explained 14.96% of the total variance in the MSMS scale. Four items *job security*, *social status/prestige*, *high income* and *proposed by parents* has been loaded on factor 2. Internal consistency of this factor 2 was checked (Cronbach’s alpha = 0.613 increases to 0.660 by dropping item *proposed by parents*). This item was eliminated so the final sub-scale contains three items.

Factor 3: Humanitarian factor of motivation. *Desire to help others*, *desire to give back to their home community or country* and i*nterest in medicine as a subject matter* has been loaded on factor 3. Internal consistency of this factor 3 was checked (Table 3). By dropping item i*nterest in medicine as a subject matter*, Cronbach’s alpha increased from 0.599 to 0.699. Because this item was eliminated, the final sub-scale contains two items. Factor 3 is named as “humanitarian factor of motivation” and explained 13.04% of the total variance.

No item cross-loaded on more than one subscale with loading greater than .40. The Cronbach’s alpha coefficient, convergent and discriminant validity for three subscales are shown in Table 3. The frequencies of responses for various items had been reported in Table 4.

**Table 4:** Category frequencies for each item under three subscales.

Factors	Category frequency				
	1	2	3	4	5
**Scientific**					
Opportunities to travel and work internationally	148	82	144	137	125
Ability to use new cutting-edge technologies	98	76	149	189	124
Research opportunities	87	68	129	211	141
**Societal**					
Job security	49	38	79	313	157
Social status	31	31	50	290	234
High income	113	96	111	220	96
**Humanitarian**					
Desire to help others	14	7	52	350	213
Desire to give back to community	17	26	95	326	172

**Fig 2:**
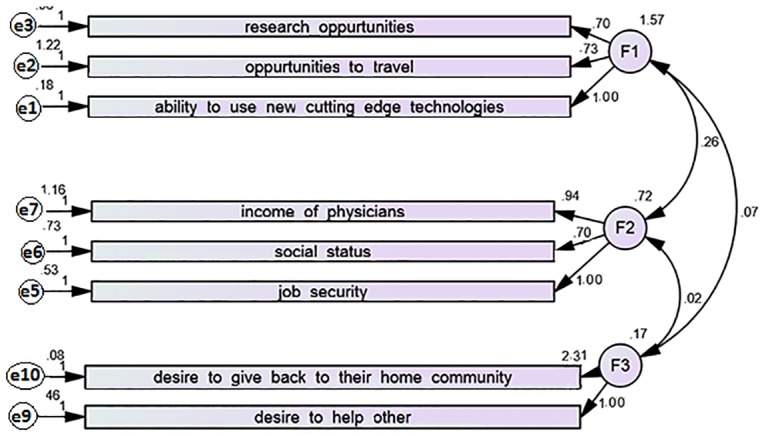
Measurement model obtained in Confirmatory factor analysis for MSMS scale.

CFA was conducted on the factors (subscales) obtained in EFA. The CFA measurement model is presented in Fig 2. Per conventional path diagram notation, the latent variables are depicted by circles and indicators by squares or rectangles. CFA goodness of fit indices obtained are CFI = .925, GFI = .959, and RMSEA = .081which suggested an acceptable model fit.

## Discussion

In the current study, we developed a MSMS questionnaire for measuring reasons of motivation to select medical study by medical students in North India. Scientific factor, which consider the interest in medicine as a scientific field; societal factors, which points towards the societal expectations and pressures; and humanitarian factors, which takes into account the intrinsic need to serve the poor and the needy emerged out as three major dimensions to measure the motives behind student’s choice of medicine. These dimensions, however, cut across the traditional dichotomy between intrinsic and extrinsic motivation and highlight instead a novel distinction. These dimensions have never been consolidated into a single comprehensive tool in earlier studies. These three subscales with an 8 item scale is a valid and reliable tool and therefore could be used to study the intentions of medical students to join medicine in India and other similar settings.

Various countries or regions have developed a variety of instruments to measure reasons of motivation to select medical study, however, they either were not standardized or focused on different goals and populations. For example, Agyei-Baffour [13] used a questionnaire on medical students of Ghana to assess the role of intrinsic and extrinsic motivation on their willingness to work in rural areas, rather than measuring motivational factors to join medical study. Further, the scale was not validated and categorization into broad heads of scientific, societal and humanitarian factors was not done. Some other tools such as the Academic Motivation Scale (AMS) by Vallerand et al. [36, 37, 38], Maslach Burnout Inventory-Student Survey (MBI-SS) containing Exhaustion scale [39] and Strength of Motivation for Medical School (SMMS) questionnaire [40, 41] for evaluation of strength of motivation of students for medical study exist in literature but all of them have been validated in western countries and due to cultural differences these are difficult to apply in developing countries like India.

There is very limited literature on the factors underpinning medical students’ choice for medical study. The self-determination theory postulates that the factors for motivation are dichotomised into intrinsic and extrinsic, which can interchange depending on various factors. With regards to intrinsic motivation, some studies [10, 13, 31, 33, 54] report that ‘serving their country’ and ‘serving humanity’ are amongst the strongest reasons for choosing medical study. In contrast, a study conducted in Ahmedabad, India reported that only 8% of students wanted to serve the poor and the main intrinsic motivator to select medical study that emerged out in this study was interest in medicine [34]. With regard to extrinsic motivation, few studies [55–59] reported that prestige, money, and personal development are important factors in career decision-making among medical students. A study by Shahab et al. [31] in Pakistan reported that medical students choose medicine because their parents wanted them to be doctors and because of their interest in medicine. A study conducted by Greenhalgh et al. [60] in UK highlighted that students belonging to higher socioeconomic status had more intrinsic motivation for seeking admission to medical college. In contrast, the students from lower socioeconomic class focused more on extrinsic rewards and higher expected income on becoming a doctor. In the present study, the three factors of motivation viz. scientific factors, societal expectations and humanitarian needs were extracted, thus offering a new perspective that goes beyond the traditional distinction between intrinsic and extrinsic motivators.

## Limitations

The conclusion of this study should be seen in light of a few design limitations. Our sample consists of students from medical colleges of three states of the country of India which may not necessarily represent the entire medical student population of the country. Moreover most of the students are Hindu, unmarried and younger (lying in range 20 to 23 years), thus representing the outcomes for this particular group. But these results can be generalized because in India, the majority of the population belongs to the Hindu community. Further, all the motivation items were equally weighted and some might have not been included in the questionnaire, despite best efforts of the researchers through extensive literature review and adopting group consensus methods.

While generalizing the results of this study, it should be taken into account that our study does not provide in-depth understanding for low motivation of MBBS students. Hence, it is recommended that further exploratory, mixed method studies, with focus group discussions or interviews, should be done to collect in-depth information for exploring the reasons of varying levels of motivation among medical students.

Considering huge shortage of doctors especially in rural areas, a further research is also needed to be carried out amongst high school students to extract the factors that motivate or de-motivate them to choose medical study. Further, it needs to be explored that out of those who completed their medical study, what proportion of them wish to stay in India and to work in rural areas. The need for such study is especially important in current scenario where there is dearth of students opting medical study resulting in shortage of physicians in India.

## Conclusions

To the best of our knowledge, this is the first instrument for measuring the motivation to choose medicine by medical students that has been developed and validated in India. The study also proposes a more salient motivational taxonomy that cuts across traditional distinction between intrinsic and extrinsic factors, and that takes into account the scientific appeal of medical study, the social pressures and expectations of family and friends, and the humanitarian drive, specifically to give back to their own community. The relevant recommendations can be made for practical guidance to policy-makers on how to design, implement and evaluate policy to motivate students to choose medical study. This will in turn strengthens the existing capacity of health care systems. We propose that this instrument should be applied in other populations of developing countries with shortages of medical doctors in rural areas to undertake context specific policy measures.

## Supporting Information

S1 File Socio-demographic profile and items for selection of medical studies of medical students in India. (SAV)
